# The effect of sports massage on state anxiety in elite soccer players

**DOI:** 10.3389/fpsyg.2025.1724892

**Published:** 2025-11-28

**Authors:** Mine Taskin, Erol Korkmaz, Maya Budak, Ibrahim Halil Sahin, Halil Taskin

**Affiliations:** 1 Beyşehir Ali Akkanat School of Applied Sciences, Selcuk Universitesi, Konya, Türkiye; 2 Konyaspor Football Club, Konya, Türkiye; 3 Ministry of National Education, Erzincan, Türkiye; 4 Istanbul Esenyurt Universitesi, Esenyurt, Türkiye; 5 Sport Science Faculty, Selçuk University, Konya, Türkiye

**Keywords:** sports massage, state anxiety, pre-match anxiety, elite soccer players, psychological intervention, performance enhancement

## Abstract

**Background:**

A sports massage represents an intervention that is frequently used in sports for that purpose, to recover and prepare an athlete for the following match. Studies that link massage and psychological mechanisms are scarce. In the athletic and recreational sport population, most of the studies confirmed a positive correlation between a massage and the improvement of different psychological states. This study was conducted to evaluate the effectiveness of Sports Massage intervention on pre-match anxiety.

**Methods:**

A total of 26 elite soccer players participated in this study and were pretest and posttest by using the Quasi-Experimental study design, quantitatively. The instrument used in this study was State–Trait Anxiety Inventory (STAI). The state anxiety inventory was administered to the soccer players 30–45 min before the competition. There were two phases of instrument measurement in this study namely pre-massage (pretest) and post-massage (posttest).

**Results:**

Binary logistic regression analysis was performed. There was a significant difference in the between pre massage anxiety and post massage anxiety (*p* < 0.05). Thus, the logistic regression model was effective in predicting the relationship between massage intervention and with reduction of anxiety. In a complementary logistic model, the odds of high anxiety were lower after massage (OR = 0.85; 95% CI) and ROC analysis yielded AUC = 0.76. Post-massage STAI-State scores were lower than pre-massage (mean ± SD: 28.35 ± 5.36 vs. 35.15 ± 7.70); we report the effect size (Cohen’s d value = 1.03) and 95% CI for interpretability.

**Conclusion:**

The study found that pre-match massage significantly affected the state anxiety of elite soccer players. Soccer players who had a massage showed 0.849 times smaller anxiety scores than did the players who had not. This finding suggests the efficacy of sports massage as an effective intervention to reduce pre-competition anxiety in elite soccer players.

## 1 Introduction

Massage has been defined as a systematic form of touching the soft tissues with palm and fingers for the purpose of promoting health and wellbeing (Moyer et al., 2004). According to Field et al. (2005) massage allows an increase in artery, vein, and local bloodstream and stroke volume. Inhibit the pain and improve the defecation. Serotonin and dopamine increase, and cortisol and awareness decrease. It is believed that massage to athletes is beneficial via biomechanical, physiological, neurological, and psychological mechanisms (Weerapong et al., 2005). Thus, athletes use numerous physical routines before their exercise or sporting competition. Some of these, like stretching and massage, may also contribute to positive mental states. Excess physical activity causes tension and muscle stress, so in relation to sports massage will release it. Because sports massage has been suggested as a tool to prepare an athlete for competition, increase athletic performance, as a treatment to assist an athlete in recovering after exercise or competition, and as a treatment for sports-related musculoskeletal injuries (Cassar, 2004; Holey and Cook, 2003; Galloway and Watt, 2004), it is clear why it is often appended to clinical trials, or as part of a recovery program in sports training clinics. However, sports massage is a type of massage that athletes commonly used to keep their physical health in shape. The benefits of sports massage on blood circulation are increasing muscle tissue temperature, increasing Muscular elasticity, stimulating breathing, and reducing or eliminating nervous tension to reduce pain (Ripai and Graha, 2018). Sports massage has been accepted in many parts of the world and is an ongoing form of therapy. According to what has been scientifically proven, using sports massage therapy before and after exercise can increase performance, promote recovery and prevent injury (Gasibat et al., 2017). Massage is thought to help elongate the muscles, and improve the compliance, allowing for better range of motion to allow the body to perform better. Athletes are often shrouded in pre-competition anxiety before participating in any actual tournament. This is due to physical or psychological problems, fear of defeat, feelings of inadequacy, loss of control and guilt (Amasiatu and Uko, 2013; Pa et al., 2020). The decrease in people’s performance is one of the commonest mental disorders of human daily life known as anxiety. As one of the psychological factors, anxiety should be considered in sports psychology to support athletes to perform well (Weinberg and Gould, 2021). Pre-competition anxiety among athletes usually occurs within 24 h until the time of the competition (Rosli et al., 2015). A significant factor preventing players from performing in sports is the pressure for success, which also increases a player’s anxiety. State anxiety reflects a transitory emotional state or a condition that is characterized by subjective, consciously perceived feelings of tension and apprehension, and heightened autonomic nervous system activity. State anxiety can be defined as the sum of these singly conscious perceived feelings of tension and apprehension and autonomic nervous system activity. On amendment of anxiety by instructing the players with a pressure laden instruction, performance deteriorated in terms of reduction of shooting accuracy and increase in the response time under high-threat conditions (Wilson et al., 2009; Wood and Wilson, 2010; Behan and Wilson, 2008; Vine and Wilson, 2010). In the competitive sports situation that requires a success result, it is important to reduce, as much as possible, the debilitating effects of anxiety from performance. According to theoretical model of the expected mechanisms of massage, possible mechanisms of massage were biomechanical, physiological, neurological, and psychological effects. Psychological effects increased the relationship between body and mind. Also, it increased relaxation and reduced anxiety (Weerapong et al., 2005). Brummitt (2008) reported that additional studies examining the physiological and psychological effects of sports massage are needed to assist sports physical therapists in developing and implementing clinically meaningful, evidence-based programs or treatments. While there are numerous studies covering anxiety and massage, research using massage to reduce pre-competition anxiety is quite limited. In addition, no study was found to investigate the effect of massage on the pre-match state anxiety of elite soccer players. It is hypothesized that most soccer players use massage, with pre-match massage likely the most popular choice by all levels of soccer players, due to its as part of the warm-up and psychological relaxation. Therefore, the aim of this study was to examine the effects of pre-match massage on pre-match state anxiety of elite soccer players.

## 2 Methods

### 2.1 Subjects

Twenty-six men elite soccer players were examined in total. All soccer players in this study voluntarily participated. The mean (SD) age was 28.88 ± 4.51 years, height was 181.81 ± 6.64 cm, and weight was 78.95 ± 6.74 kg for the 26 soccer players. These soccer players were playing in Turkish Super League team which is named Konyaspor. All players were elite soccer players competing actively in national or international competitions. In addition, players had to be in good physical health and not have any acute or chronic injuries that could prevent massage therapy from being functional. Soccer players had a minimum of 10 to a maximum of 15 years in the game of competitive soccer. Before conducting the experiment, all soccer players were informed of the risks of the study and gave informed consent. The research was conducted with the knowledge and approval of the faculty administration. Inclusion and exclusion criteria; The study included soccer players who regularly trained under the guidance of a coach at least 5 days a week and played one official match. Players with recent physical injuries or health problems were excluded.

### 2.2 Procedures

Data were collected through the Spielberger Anxiety Inventory State–Trait Anxiety Inventory (STAI) to examine the conjecture of the present investigation. The state anxiety inventory was administered to the soccer players 30–45 min before the competition. Following the inventory administration, the soccer players were given a sports massage and when the massage was over, the state anxiety inventory was administered again and the soccer players went out to warm up for the match on the football field. Sports massage protocols in research vary a great deal, thus, determining the actual true effects becomes a challenging task. Robertson et al. (2004) recommend that leg massage last 10 min, with most massages requiring 10–30 min for effectiveness. In our study, Robertson et al. (2004) recommended massage protocol was applied.

#### 2.2.1 Massage protocol

Five club physiotherapists delivered the protocol. Each treated one player at a time (starting XI first, then substitutes) following an identical sequence: prone 5 min for posterior legs → supine 5 min for anterior legs, with mineral oil (~10 mL per region; ~40 mL total), per Robertson et al. (2004). Effleurage and friction techniques were used in the massage intervention. Effleurage: Whole hand two handed, centripetal and multidirectional. Friction: The application of deep pressure to a specific tissue using all fingers or just the thumb and performing a controlled friction movement on the tissues.

#### 2.2.2 Spielberger anxiety inventory state–trait anxiety inventory-state

Data collection included Spielberger anxiety inventory State–Trait Anxiety Inventory (STAI) (Spielberger, 1983). State anxiety inventory was designed by Spielberger in 1970 and was revised in 1983. This test has two scales: state anxiety and trait anxiety. Each of these scales has 20 items with a Likert scale of 1–4. In the current study, the section of state anxiety was used which consists of 20 self-descriptive statements for the state anxiety scale. The state anxiety scale evaluates the soccer players’ state of anxiety at the moment of the pre and post competitive interview, measured on a four-point Likert-type scale; no = 1, a little = 2, a lot = 3, totally = 4 (Ferber and Makhoul, 2004).

We administered the STAI-State (20 items; 4-point Likert) and followed Spielberger scoring (total 20–80, higher = greater state anxiety). The validated Turkish version was used (cite). STAI-State was completed 30–45 min before kick-off and immediately after the 10-min massage (before warm-up).

### 2.3 Statistical analyses

Statistical analyses were performed using IBM SPSS statistics software (version 27). Data is presented as mean ± SD. The normality of the variables was assessed with the Kolmogorov–Smirnov test. The reliability of the performance tests was analyzed using the intraclass correlation coefficient (ICC), and the coefficient of variation (CV) for absolute reliability. Ninety-five percent confidence intervals (CIs) were calculated for ICC and CV. The dependent variable in the model was whether massage or not massage are used. For the regression analysis, binary logistic regression analysis was assessed to determine the effect of massage on state anxiety. As a complementary analysis, we fit a binary logistic regression modeling the odds of high anxiety by condition and report odds ratios and AUC (95% CI).

## 3 Results

STAI-State scores were lower after massage than before (28.35 ± 5.36 vs. 35.15 ± 7.70). For interpretability we provide the effect size and 95% CI. In a complementary model, the odds of high anxiety were lower post-massage (OR = 0.85), and ROC AUC was 0.76.

The 26 soccer players (age = 28.88 years, SD = 4.51) participated in the study. Their physical characteristics (height, weight) and state anxiety scores were described in Table 1. In terms of age distribution, the mean age of the players was 28.88 years, and the standard deviation was 4.51 years, showing a relatively homogenous group (the mean age and standard deviation). The mean heights were 181.81 cm, and the body weights were 78.95 kg, with a standard deviation of 6.64 cm and 6.74 kg, respectively. These values indicate that the sample comprised well-conditioned athletes of similar physical attributes. The state anxiety score before massage was 35.15, and diminished down to 28.35 for after the massage.

**Table 1:** Means and standard deviations of the physical characteristics and state anxiety score of the soccer players (*n* = 26).

Variables	n	Mean ± SD	95% confidence interval mean	
			Lower	Upper
Age (years)	26	28.88 ± 4.51	24.145	27.614
Height (cm)	26	181.81 ± 6.64	179.258	184.361
Body weight (kg)	26	78.95 ± 6.74	76.361	81.539
State anxiety (score)	Not massage	35.15 ± 7.70	32.705	38.145
	Massage	28.35 ± 5.36	25.616	31.084

The 2 × 2 correct classification percentage using the logistic regression model that predicts state anxiety level from massage intervention is presented in Table 2. Regarding the state anxiety variable, 17 out of 26 massaged soccer players were predicted correctly in this model, while 17 out of 26 people who did not get massaged were predicted correctly. As a result, we obtained an overall accuracy of 65.4%.

**Table 2:** 2 × 2 correct classification percentage for logistic regression model in state anxiety.

	Predicted		
Observed	Not massage	Massage	% correct
Not massage	17	9	65.4
Massage	9	17	65.4

**Figure 1:**
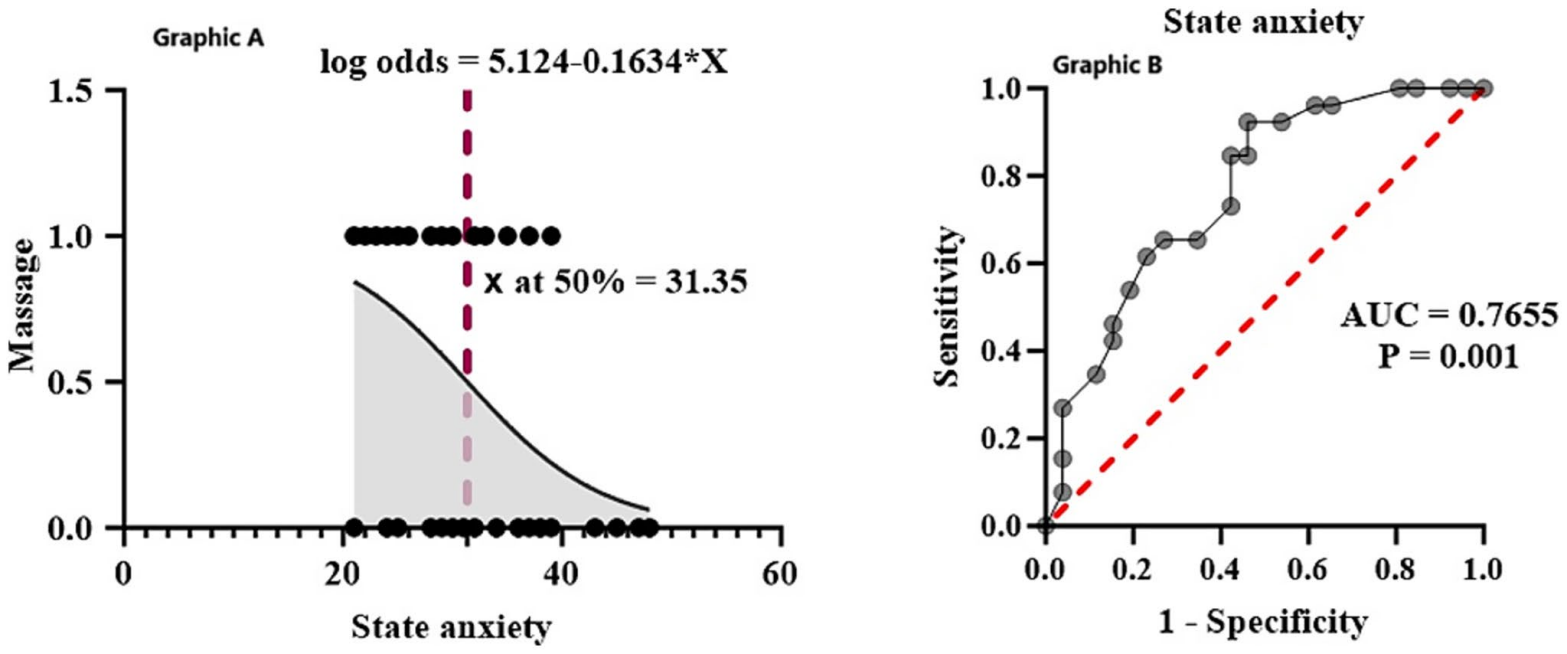
When the effect of independent variables on the dependent variable is examined, one unit change in massage affects state anxiety score by 0.1634 units **(A)**. Testing the predictive power of state anxiety using receiver operating characteristic curve. Area under the curve (AUC) and *p* value are indicated. The predictive power of state anxiety variable individually was evaluated using the area under a ROC curve (AUC). Analysis was performed non-massage (*n* = 26) and massage (*n* = 26) volunteers **(B)**.

Table 3 shows the values for the model coefficients, the log odds, odds ratio, confidence interval and *p* values for the Logistic Regression output. The odds ratio of the key predictor, massage intervention, was 0.849; that is, by receiving a massage, the players’ anxiety levels were reduced by 0.849-fold versus not receiving a massage. This was further confirmed by the fact that the *p*-value of 0.002 showed that the relationship between massage and anxiety reduction was statistically significant. In addition to the hypothesis regarding the relationship between receiving a pre-match massage and reducing the state anxiety score by one unit, the regression analysis confirmed this as a case, with the state anxiety score being declined by 0.163 units for a unit change in receiving a massage. The findings from these studies emphasize the efficiency of sports massage to decrease state anxiety in elite soccer players. They indicate its capacity as a pivotal psychological resource during elite sports performance management (Figure 1).

**Table 3:** The output of the logistic regression: Log odds, odds ratio, and confidence intervals (95% CI) for the odds ratio and *p*-values of the coefficients.

Model coefficients: state anxiety		95% CI					
Predictor	Estimate	Lower	Upper	SE	Z	*p*	Odds ratio
Intercept	5.124	1.852	8.395	1.669	3.07	0.002	167.947
Massage	−0.163	−0.267	−0.060	0.053	−3.10	0.002	0.849

## 4 Discussion

In this study, we examined using massage whether the massage influences state anxiety level in the pre soccer match. The main finding of the study was that a 10-min pre-match massage administered by club physiotherapists prior to a league match provided a significant benefit in terms of reducing state anxiety levels. The results revealed a substantial reduction in the state anxiety scores in response to massage. The decrease in anxiety levels reinforces the hypothesis that sports massage has a beneficial influence on the reduction of pre-competition anxiety in elite soccer players. This is an important result, given that elite soccer players have high expectations, intense pressure and high levels of psychological stress prior to important matches. In a study conducted on amateur soccer players, the effect of massage on pre-match anxiety was examined. It was reported that massage had a reducing effect on pre-match state anxiety and should be one of the methods used to reduce pre-match anxiety (Arslan et al., 2010). Previously a study indicated that massage enhanced positive effect (*p* < 0.05), lowered self-reported perceptions of physical symptoms (*p* < 0.05), and decreased perceived physical effort (*p* < 0.05). It confirms the positive, and consequently motivational, impact of a brief massage session on exercise behavior (Szabo et al., 2008). In Goats (1994) illustrates that a 10-min pre-match massage increases positive effect and decreases perceived physical symptoms. The decrease in physical symptoms may be attributed directly to the therapeutic effects of massage on blood flow, connective tissues, muscle, and nervous system. A study by Shuiabu and Bello (2019) reported that massage before World Cup matches reduced anxiety in Nigeria national team soccer players. A study conducted on 14 elite Malaysian tennis athletes reported that pre-competition sports massage had a significant impact on cognitive anxiety, somatic anxiety, and self-confidence. It also highlighted that pre-competition massage is an effective approach to helping elite tennis players cope with anxiety (Pa et al., 2021). Pre-competition anxiety is thought to have a significant negative impact on tennis forehand and backhand accuracy in Malaysian university tennis players, and massage is thought to be one of the best approaches to overcome this (Pa et al., 2024). A previous study found that massage therapy significantly reduced wrestlers’ depression, anxiety, and stress levels (Zadkhosh et al., 2015). The results of our research align with findings from previous studies on the benefits of massage in sports settings.

### 4.1 Limitation

One of the main limitations of this study was that football players’ out of competition general anxiety levels could not be compared with competition anxiety. Other limitations of the study, this study only recruited elite soccer players as experimental subjects, so the findings may not be applicable to athletes doing individual sports and amateur. We considered only soccer players between 18 and 33 years of age to have a homogeneous sample based on their physical capabilities and recovery potential. Although previous research has shown some gender differences in how male and female athletes respond to anxiety-provoking situations and relaxation interventions, only male soccer players participated in this study. Therefore, it is recommended that subsequent researchers extend the study to examine changes in pre-match state anxiety or explore other sport branches and age groups to help fill in the research gaps.

## 5 Conclusion

The results of this study suggest that pre-match massage is quite beneficial in calming anxious players. Given this, this study provides strong evidence that sports massage effectively reduces pre-match state anxiety among elite soccer players. In support of consideration for massage as part of an athlete’s pre-match preparation, the statistically significant reduction in anxiety scores and the predictive accuracy of the logistic regression model validate that massage should be part of an athlete’s pre-match preparation. Thus, the logistic regression model was effective in predicting the relationship between massage intervention and with reduction of anxiety. This seems to indicate that massage can be a good predictor of the level of anxiety in the athletes just prior to a competition, hence supporting the inclusion of sports massage as a psychological intervention in competitive settings. However, this study has demonstrated the usefulness of sports massage as a potential, valuable, both non-invasive and effective alternative for the management of pre-match anxiety by elite soccer players. In this one-group pre–post setting, a brief pre-match leg massage was associated with lower immediate STAI-State scores; causal inference is limited in the absence of a control/sham condition. Future studies with larger sample sizes will be important for the accuracy of the results. It is also recommended that future studies examine not only state anxiety levels but also individual performance metrics of the soccer players to determine whether massage helps more than just alleviating state anxiety.

## Data Availability

The raw data supporting the conclusions of this article will be made available by the authors, without undue reservation.
